# Assessing the impact of exome sequencing on diagnostic yield in a large cohort of Brazilian patients

**DOI:** 10.1590/1806-9282.20250086

**Published:** 2025-08-15

**Authors:** Aline Cristiane Planello, Thereza Loureiro, Dayse Alencar-Cupertino, Diana Bermeo, Joselito Sobreira, Mireille Gomes, Luciana Santos Serrao de Castro, Darine Villela, Cristovam Scapulatempo-Neto, Nara Sobreira

**Affiliations:** 1 Diagnósticos da América S.A.– São Paulo (SP), Brazil.; 2 Instituto de Ensino e Pesquisa DASA – São Paulo (SP), Brazil.; 3 Johns Hopkins University, School of Medicine, McKusick-Nathans Department of Genetic Medicine – Baltimore (MD), United States.

**Keywords:** Genomic medicine, Sequence analysis, Exome sequencing, Genetic testing, Genetic counseling

## Abstract

**OBJECTIVE:**

This study aimed to evaluate whether the increased use of exome sequencing as a primary next-generation sequencing test provides a higher diagnostic yield compared to multi-gene panels for various clinical indications within a Brazilian cohort.

**METHODS:**

We retrospectively analyzed test results from 3,025 patients who underwent single-gene tests, multi-gene panels, and exome sequencing in our laboratory. The results were categorized as positive, inconclusive, or negative, with clinical indications including neurodevelopmental, late-onset neurological, neuromuscular, syndromic, skin/hair, hematological/immunologic, ophthalmologic, inborn errors of metabolism, skeletal, cardiovascular, reproductive planning, cancer, genitourinary, gastrointestinal, and hearing disorders.

**RESULTS:**

Of the 3,025 next-generation sequencing-based tests, 420 were single-gene tests, 1,158 were multi-gene panels, and 1,447 were exome sequencing. Although multi-gene panels were initially the most frequently ordered test, exome sequencing currently represents 62% of all next-generation sequencing-based tests performed in our laboratory. Exome sequencing had the highest detection rate (32.7%), but also the highest inconclusive rate. Among the clinical indications, when stratified by exome sequencing, skeletal and hearing disorders had the highest diagnostic yield, corresponding to 55 and 50%, respectively.

**CONCLUSION:**

This study emphasizes the diagnostic usefulness of exome sequencing for complex genetic disorders in postnatal diagnosis. However, the expansion of exome sequencing also presents challenges, including an increased rate of inconclusive results due to variants of uncertain significance. Broader availability of exome sequencing must be accompanied by additional resources to address these challenges effectively.

## INTRODUCTION

Next-generation sequencing (NGS) technology has been rapidly incorporated into clinical laboratory testing. Current applications include the detection of either germline or somatic variants, and laboratories may focus their analysis on single-gene tests, multi-gene panels, and exome or whole-genome sequencing. While single-gene tests analyze only one gene and are used to confirm or rule out a specific diagnosis, multi-gene panels analyze several genes at once and can identify mutations that may increase a patient's risk of a specific disease. Increasing the genetic tests’ resolution, exome sequencing (ES) evaluates all exons (the protein-coding regions of genes), and whole-genome sequencing interrogates the entire genome. In general, two tests are more likely in the diagnostic setting when we consider using an NGS-based test: a multi-gene panel related to the patient's phenotype sequencing or ES in case the patient presents with an undiagnosed complex phenotype affecting multiple organs and/or had a previous negative multi-gene panel result^1^. While the clinical utility of multi-gene panels has been demonstrated for various conditions^2^, the development of new NGS platforms that offer ultra-high throughput, scalability, and speed has changed the clinical scenario, and ES is already becoming the most common molecular diagnostic test in several medical centers^3^. Nonetheless, the diagnostic yield of an NGS-based test varies and depends on several factors, including the distribution of pathogenic variants associated with genes that explain the patient phenotype, a comprehensive clinical evaluation, the expertise of genomic specialists to identify clinically relevant variants and properly assess their pathogenicity, and the available evidence to classify the candidate variants including the knowledge of allele frequency in the population of interest.

Despite Europe and the USA adopting early the ES as first-tier diagnostic testing for being considered the most cost-effective test, especially for constitutive diseases in postnatal diagnosis^3,4^, the high costs associated with implementing such technology created a significant disparity among other countries^5^. In Brazil, most patients with access to an NGS-based test have private health insurance and are concentrated in the country's Southeastern region^6,7^. In contrast, in the public system, an NGS-based test is provided only for a few patients as part of research projects at school-hospitals affiliated with universities. The DASA laboratory is located in São Paulo and has developed a vast portfolio of genetic tests to attend to the private sector, offering more than 700 service units nationwide. In our clinical routine, we have validated single-gene tests, multi-gene panels, and exome and whole-genome sequencing for various inherited diseases and somatic variants. With ES orders increasing annually in our clinical routine, it allowed us to assess a large cohort of Brazilian patients referred to molecular investigation, representing a genetically diverse and underrepresented population. In this study, we aimed to assess whether the increased use of ES as a primary diagnostic approach over the years has led to a higher diagnostic yield in comparison to single-gene and multi-gene panel testing. We hypothesized that ES, with its broad genomic coverage, would enhance the identification of pathogenic variants, particularly for complex conditions, and improve diagnostic outcomes in routine practice.

## METHODS

### Ethical approval

This study was approved by the Ethics Committee from Hospital 9 de Julho (CAAE: 55817821.3.0000.5455), and informed consent was obtained from all patients for genetic testing. Ethical Approval date: 21/02/2022.

### Study design

This retrospective observational study collected NGS-based test results from patients investigated in our laboratory between 2018 and 2021. In total, 3,025 patients were referred for molecular investigation due to a suspected genetic disease. The genetic testing was performed upon a medical request. Clinical indications were grouped into (1) neurodevelopmental disorders, (2) late-onset neurological disorders, (3) neuromuscular disorders, (4) syndromic disorders, (5) skin/hair disorders, (6) hematological/immunologic disorders, (7) ophthalmologic disorders, (8) inborn errors of metabolism (IEM), (9) skeletal disorders, (10) cardiovascular disorders, (11) reproductive planning, (12) cancer, (13) genitourinary disorders, (14) gastrointestinal disorders, and (15) hearing disorders. NGS methods include single-gene tests, multi-gene panels, and ES. Detected variants were classified for their clinical impact according to the American College of Medical Genetics (ACMG) guidelines^8^.

### Data analysis and statistics

NGS-based test results were categorized as positive, inconclusive, or negative. A positive result was determined by the presence of heterozygous pathogenic or likely pathogenic (P/LP) variant(s) in a gene associated with an autosomal dominant disease, homozygous or compound heterozygous P/LP variants in a gene associated with autosomal recessive disease, or hemizygous P/LP variant in a gene associated with an X-linked recessive disorder. Additionally, an inconclusive result was defined as heterozygous and hemizygous variants of uncertain significance (VUS) in genes known to cause autosomal dominant or X-linked recessive diseases, respectively, or homozygous and compound heterozygous VUSs in genes known to cause autosomal recessive diseases, or as the combination of a VUS and a P/LP in a gene known to cause an autosomal recessive disease. Compound heterozygosity was defined by the presence of two heterozygous variants in the same gene, regardless of whether they were in cis or trans, since we only have sequencing information of probands. A negative result was determined by the presence of benign or likely benign (B/LB) variant(s). Secondary findings in ES, i.e., a genetic variation that may contribute to a disease, but is not related to the primary purpose for the genetic testing referral, were reported when the patient or their parent/guardian opted in as indicated in the informed consent. In this case, only variants classified as P/LP in the ACMG actionable genes were reported^8^.

Statistical analysis was performed using R software (version 4.2.2). Data was summarized using descriptive statistics, presenting categorical variables as absolute frequency and percentages. For comparison between diagnostic rates of NGS-based tests, we employed the χ^2^-test. The significance level was set as a p-value lower than 0.05.

## RESULTS

Of the 3,025 patients’ NGS tests performed in our laboratory routine, 420 were single-gene tests, 1,158 were multi-gene panels, and 1,447 were ES. The median age of the patients at diagnosis was 12 years old (IQR 6–37; range 2–91 year), and 57% were male and 43% female. From 2018 to 2020, the most frequently ordered tests were the muti-gene panels, followed by ES. However, in 2021, ES became the most frequently ordered test, representing 55.3% of the total NGS tests performed that year (Table 1). In general, the primary clinical indications for genetic testing were syndromic and neurodevelopmental disorders, ES being the most frequently prescribed test for these two conditions, corresponding to 76.2 and 74%, respectively (Table 2).

**Table 1:** Next-generation sequencing-based tests ordered per year and by test type.

Year	ES n (%)	Multi-gene panel n (%)	Single gene n (%)
2018	39 (30.5)	69 (53.9)	20 (15.6)
2019	107 (32.6)	171 (52.1)	50 (15.3)
2020	262 (38.0)	285 (41.4)	142 (20.6)
2021	1,039 (55.3)	633 (33.7)	208 (11.0)

ES: exome sequencing.

**Table 2:** Distribution of next-generation sequencing-based tests according to clinical indications.

Clinical indication	ES (%)	Multi-gene panel (%)	Single gene (%)
Syndromic (n=793)	76.1	13.9	10.0
Neurodevelopmental (n=803)	74	22	4
Gastrointestinal (n=10)	50	50	0
Late-onset neurological (n=214)	33	54	13
Reproductive planning (n=46)	28	17	54
Genitourinary (n=52)	26.9	50.0	23.1
Neuromuscular (n=110)	26	59	15
Skin/hair (n=39)	25.7	53.8	20.5
Hematological/immunologic (n=109)	24.8	47.7	27.5
Ophthalmologic (n=38)	21	74	5
Hearing (n=20)	20	80	0
Cancer (n=63)	15.9	71.4	12.7
Cardiovascular (n=85)	14.1	40.0	45.9
Skeletal (n=197)	10.2	76.6	13.2
Inborn errors of metabolism (IEM) (n=446)	6	69	25

ES: exome sequencing.

Overall, 28.4% of the patients (860/3025) received a positive NGS test result. Taking into account the diagnostic yield, ES had the highest detection rate (32.7%), and it also had the highest inconclusive and the lowest negative rates (p<0.001). The ES diagnostic yield remained relatively constant during the years, but the negative results decreased. Among the clinical indications, skeletal and hearing disorders had the highest diagnostic yield. When stratified by ES, both conditions had a detection rate of 55 and 50%, respectively (Table 3).

**Table 3:** Distribution of diagnostic yield by next-generation sequencing-based tests, including single-gene tests, multi-gene panels, and exome sequencing.

Type of test	Positive (%)	Inconclusive (%)	Negative (%)
Exome sequencing (ES) (n=1,447)	32.7	43.9	23.4
Multi-gene panel (n=1,158)	25.0	29.3	45.7
Single gene (n=420)	23.0	11.0	66.0
**Clinical indication of ES**			
Skeletal (n=20)	55	25	20
Hearing (n=4)	50	50	0
Ophthalmologic (n=8)	38	38	25
Syndromic (n=504)	35.6	41.1	23.3
Neurodevelopmental (n=503)	33	46	21
Genitourinary (n=29)	27.6	41.4	31.0
Neuromuscular (n=12)	25	50	25
Cardiovascular (n=27)	23	54	23
Reproductive planning (n=13)	22	44	34
Hematological/immunologic (n=5)	20	40	40
Gastrointestinal (n=10)	20	40	40
Cancer (n=11)	18.6	54.3	27.1
Late-onset neurological (n=11)	18	46	36
Inborn errors of metabolism (IEM) (n=8)	18	46	36
Skin/hair (n=20)	10	20	70

Regarding the detected variants in ES, 1,956 variants were classified as P/LP or VUS, corresponding to 39 and 61%, respectively. Missense variants represented the most frequent type, accounting for 60% of the P/LP variants and 97.7% of VUS. Conversely, protein-truncating variants were more prevalent among P/LP variants, accounting for 40% while representing only 2.3% of the VUSs. Of note, the rate of secondary findings in our ES dataset was 3%.

## DISCUSSION

In this study, we demonstrated that ES had the highest diagnostic yield among other NGS-based tests, supporting its role as a first-tier diagnostic tool for complex diseases like neurodevelopmental and syndromic disorders.

Although multi-gene panels were the most frequently ordered genetic test in the first years of the NGS implementation in our laboratory, the development of new ultra-high–throughput technologies, offering sequencing cost reduction and higher resolution, rapidly changed the diagnostic scenario. Currently, ES is the most frequently ordered test, representing 62% of all NGS-based tests performed. It's important to acknowledge that since 2021, the health insurance in Brazil has approved the coverage of ES, which certainly contributed to accelerating this transition. The cost-effectiveness of ES has been extensively demonstrated, and the recommendation from ACMG is to use either exome or genome sequencing as a first-tier genetic test for patients with neurodevelopmental disorders^9^. Despite the overall diagnostic yield of ES being higher than the multi-gene panels, the rate of inconclusive results was also higher. It is expected that as we increase the test resolution, the number of VUS will also increase since more genes are being tested^10^. In fact, this is particularly relevant for the Brazilian population, which is often underrepresented in public genomic databases, directly impacting variant classification, with a proportion of VUS exceeding the average observed in other populations^11^. In our dataset, this can also be due to the proband-only sequencing. Trio testing (proband and their parents) is a better approach for ES since it improves variant classification, facilitates the identification of compound heterozygous and de novo variants, reduces the number of VUS and the analysis time, and improves diagnostic accuracy^12^. However, the high cost associated with ES makes it difficult for the trio testing approach to be authorized by health insurance in Brazil.

While ES is considered the most comprehensive diagnostic option in the postnatal setting, the test still fails to determine the genetic etiology in most patients examined^13^. Generally, the most common clinical indications for genetic testing are syndromic features and neurodevelopmental disorders, and ES is frequently prescribed for these two indications, as demonstrated in our dataset. However, when we stratified the diagnostic yield by clinical indication, these two conditions also contributed substantially to the inconclusive and negative result rates, likely due to phenotypic complexity and the involvement of poorly characterized genes. In contrast, skeletal and hearing disorders, although less frequent, yielded proportionally more positive diagnoses, suggesting a higher burden of well-established, high-penetrance variants in these phenotypes. Thus, prioritizing the test for these conditions may enhance the identification of actionable disease variants. It is worth mentioning, though, that multi-gene panels remain an accessible diagnostic choice and represent a significant proportion of genetic testing in our clinical routine. In practice, we analyze panels extracted from ES since the sequencing costs have been reduced and it's no longer cost beneficial to perform a targeted gene sequencing; the phenotype-driven panel analysis simplifies the wet lab routine and reduces the time of annotation and sequencing analysis^14^. Single-gene tests in our laboratory are recommended mainly for specific conditions such as inborn errors of metabolism, Marfan syndrome, and cardiovascular diseases.

Despite the fact that private healthcare in Brazil adopted early the NGS technology and its use increases annually, the lack of well-trained medical geneticists associated with the highly priced NGS-based tests is hampering the widespread utilization of this technology within the country. Currently, most medical geneticists and genetic services are concentrated in the South and Southeastern regions; it is estimated that approximately 30% of the Brazilian population has access to genetic counseling and a medical genetic specialist^15^. Yet, the clinical assessment and counseling of individuals at risk for genetic disorders require significant time and effort, being crucial for guiding the selection and understanding of genetic test results. Consequently, genetic testing is not a common practice in Brazil, and we observe a significant disparity in the clinical usefulness of genomic information across different ethnic groups in our population, which is well known to be highly admixed.

## CONCLUSION

Our data revealed that ES has already become the most frequently ordered test for being more cost-effective due to its higher diagnostic yield. However, it has led to an increased number of inconclusive results due to the higher frequency of detected VUS. These findings emphasize both the diagnostic value of ES in capturing a wider range of pathogenic variants and the critical need to improve resources in variant interpretation, particularly in underrepresented and admixed populations like ours. Thus, this study provides valuable insights for other laboratories aiming to integrate ES into clinical care and advocate for improved infrastructure and expertise in variant classification to maximize the clinical utility of genomic testing in postnatal settings.

## Data Availability

The datasets generated and/or analyzed during the current study are available from the corresponding author upon reasonable request.
